# Development of an LC–Tandem Mass Spectrometry Method for the Quantitative Analysis of Hercynine in Human Whole Blood

**DOI:** 10.3390/molecules23123326

**Published:** 2018-12-14

**Authors:** Salvatore Sotgia, Rhys B. Murphy, Angelo Zinellu, David Elliot, Panagiotis Paliogiannis, Gerard Aimè Pinna, Ciriaco Carru, Arduino A. Mangoni

**Affiliations:** 1 Department of Biomedical Sciences, School of Medicine, University of Sassari, Sassari 07100, Italy; azinellu@uniss.it (A.Z.); panospaliogiannis@gmail.com (P.P.); carru@uniss.it (C.C.); 2 Department of Clinical Pharmacology, College of Medicine and Public Health, Flinders University and Flinders Medical Centre, Adelaide SA 5042, Australia; rhysbmurphy@gmail.com (R.B.M.); david.elliot@flinders.edu.au (D.E.); arduino.mangoni@flinders.edu.au (A.A.M.); 3 Department of Chemistry and Pharmacy, University of Sassari, Sassari 07100, Italy; pinger@uniss.it; 4 Quality Control Unit, University Hospital of Sassari (AOU-SS), Sassari 07100, Italy

**Keywords:** aminothione, dismutatiion, LC-tandem mass spectrometry, antioxidant, zwitterion

## Abstract

Given that the peculiar redox behavior of ergothioneine involves a rapid regeneration process, the measurement of its precursor and redox metabolite hercynine could be particularly useful in assessing its role in oxidative stress or other biological processes. Thus, a LC-MS/MS method for the determination of hercynine concentrations in whole blood was developed. After lysis of red blood cells by cold water, samples were filtered on micro concentrators at a controlled temperature of 4 °C. The clear filtered fluid was then treated with diethylpyrocarbonate to derivatize hercynine for the analysis by LC-MS/MS. The derivatized analyte was isocratically separated as a carbethoxy derivative on a C18 column with a mobile phase of an aqueous 0.1% *v*/*v* formic acid and acetonitrile (95:5). Effluents were monitored by MRM transitions at *m*/*z* 270.28→95 and 273.21→95 for hercynine and its deuterated counterpart, respectively. No cross-talk between MRM transitions was observed and a good linearity was found within a range of 35–1120 nmol/L. The LOD and LOQ were, respectively, 10.30 and 31.21 nmol/L with an intraday and intermediate precision below 7%. The average hercynine concentration in whole blood from 30 healthy male volunteers (aged 77 ± 12 years) was 178.5 ± 118.1 nmol/L. Overall, the method is easy to perform, allowing a rapid and accurate assessment of whole blood concentrations of hercynine.

## 1. Introduction

A specific organic protein transporter, ergothioneine transporter (ETT), is responsible in humans for the accumulation of dietary ergothioneine (ERT; 2-mercaptohistidine trimethylbetaine) at relatively high concentrations in mitochondria, cells, and tissues normally exposed to oxidative stress and involved in the inflammatory response [1,2,3]. Despite the widespread distribution of ERT in the blood and tissues of vertebrates, its biosynthesis is limited to certain bacteria, such as mycobacteria, cyanobacteria and some fungi, including edible mushrooms [1,4,5]. Cells lacking ETT are impermeable to ERT and its uptake from the food chain appears directly related to the extent of the expression of ETT mRNA [6]. Despite the array of functions proposed for ERT, a consensus has not yet been reached on the exact biological role of this unusual hydrophilic low-molecular-weight aminothiol. The existence of ETT [2], and its evolutionary preservation, indicate that ERT might exert a useful role in the body [7]. The available evidence suggests that ERT could exert complementary effects to those of known adaptive antioxidant systems [7]. Servillo et al. demonstrated that, aside from the formation of the disulfide form, the redox behavior of ERT differs significantly from that of other alkylthiols, such as glutathione and cysteine, and of their alkyldisulfide counterparts [8]. Once formed, ergothioneine disulfide (ESSE), which is unstable at physiological pH, undergoes a progressive and spontaneous decomposition by disproportionation, rather than an enzymatic-driven reconversion to ERT. As a result, without reducing agents, ESSE can both generate hercynine (ERY; *N*,*N*,*N*-trimethyl-histidine), the main precursor of ERT biosynthesis in the producing organisms, and regenerate ERT. This unique mechanism confers an advantage, as the rapid back-formation of ERT, following the dismutation of ESSE, restarts a new oxidative cycle faster than other antioxidants. Thus, being easily reformed, ERT acts as a free source of reducing equivalents promptly available to counteract the reactive oxygen species [9]. Such a mechanism could also explain the apparently contradictory data on ERT concentrations in some inflammatory disease states [10,11]. Potentially, the fast recovery of ERT could mask possible fluctuations in biological fluids where, due to its almost exclusive intracellular distribution and poor tissue turnover [12], its physiological concentrations are relatively low and stable [13,14]. ERY, similarly to ERT, cannot be synthesized by mammals and is produced during ESSE disproportionation. In particular, the dismutation of 2 mol of ESSE regenerates 3 mol of ERT and generates 1 mol of ERY [8]. Furthermore, ETT shows a lower affinity for ERY [15] and is therefore more easily excreted by cells than ERT. Previous qualitative/semiquantitative analyses have revealed that in key human biological specimens such as urine, plasma, saliva, and whole blood, ERY concentrations are in the order of micro- to nanomoles per liter [16]. ERY has also been shown to increase in endothelial cells exposed to high glucose [17] and a significant correlation between ERT and ERY concentrations in the whole blood has been reported in healthy human volunteers administered with ERT [18]. Therefore, as the oxidation of ERT may explain the presence of ERY in biological fluids, its determination may be more useful than ERT alone in detecting oxidative stress states or other biological processes involving ERT. Thus, the aim of this study was to develop a quantitative method by liquid chromatography–tandem mass spectrometry for the measurement of hercynine concentrations in human whole blood.

## 2. Results

### 2.1. Optimization of Chromatographic Conditions

**Figure 1:**
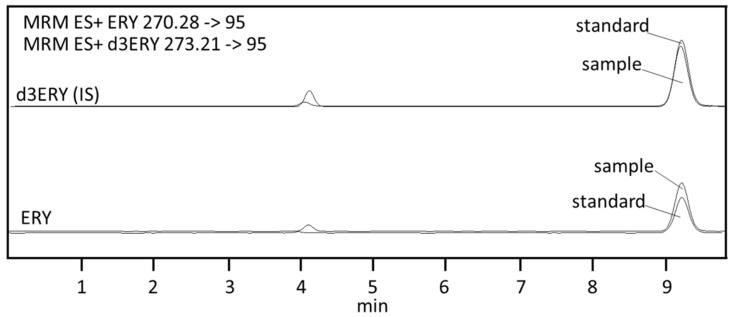
Representative chromatograms of a real whole blood sample (sample) and a 100 nmol/L standard. Solution of ERY (standard). ERY and d3ERY refer to the derivatized forms.

The effects of formic acid and acetonitrile (ACN) content in the mobile phase were evaluated in order to obtain an adequate balance between retention time, peak area (sensitivity), peak shape, and the resolution of adjacent peaks. ACN was tested in the range from 1 to 10% *v*/*v*, while formic acid was tested in the range from 0.05 to 0.2% *v*/*v*. For concentrations of ACN above 5% *v*/*v*, the retention time decreased drastically. The integration and quantitative analysis were also affected by a worsening of the peak shape and of the resolution. Conversely, for concentrations of 1–5% *v*/*v,* the ACN retention time gradually increased with a less marked loss of sensitivity and peak symmetry. Overall, the retention time of the derivatized ERY was less affected by formic acid than by ACN. At a concentration of 5% *v*/*v* ACN, the retention time was identical for concentrations of 0.1 and 0.2% *v*/*v* formic acid, while a delay of about 1 min was observed for 0.05% *v*/*v* formic acid. No significant difference was found in peak shape and sensitivity in the range of the assayed concentrations of formic acid. A mixture of an aqueous solution of 0.1% *v*/*v* formic acid and ACN (95:5) was therefore chosen as the optimum mobile phase composition. Figure 1 shows a representative chromatogram of a 100 nmol/L standard solution of ERY and a whole blood sample.

### 2.2. Optimization of the Derivatization Procedure

**Figure 2:**
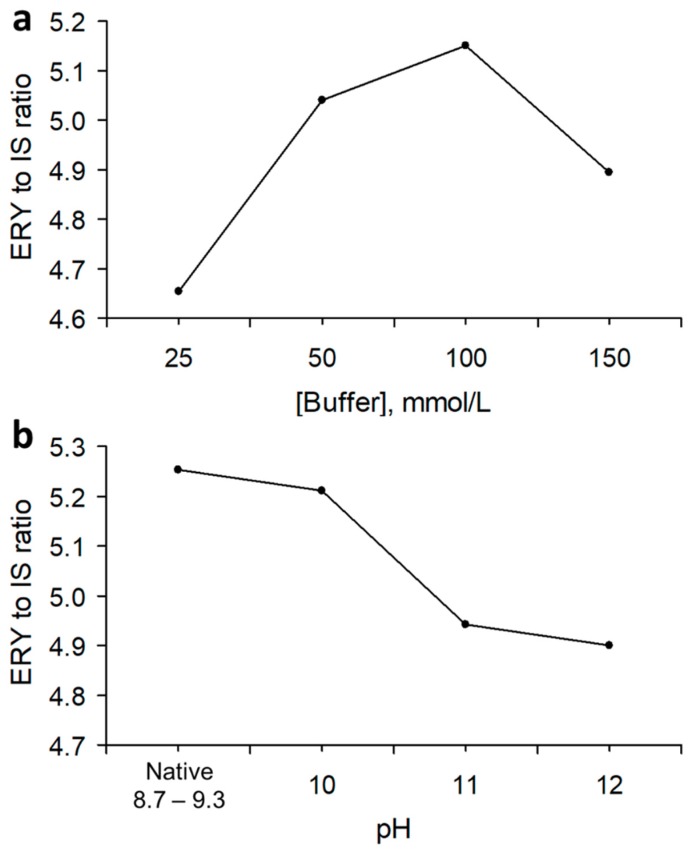
Effect of the (**a**) buffer concentration and (**b**) pH on the rate of the derivatization reaction between DEPC and ERY.

Some rough tests with different buffer and pH solutions were performed and subsequently, sodium phosphate dibasic heptahydrate proved to be a better choice in terms of sensitivity. Thus, the latter was further tested in the range of 25–150 mmol/L. In this range, the buffer had a native pH of 8.7–9.3 and the effect of the buffer concentration was initially evaluated at these pHs. As shown in Figure 2a, for a concentration of phosphate buffer of100 mmol/L, the rate of derivatization expressed as the peak area ratio between ERY and internal standard (IS) was the highest and, therefore, this buffer concentration was used to check the influence of the pH. The latter was evaluated in the range of 8.7–9.3 to 13. At a pH of 13, derivatization did not occur while, as shown in Figure 2b, the peak area was the greatest at a pH of 8.7–9.3. However, at the native pH, which is buffered around 7.4–7.8, occasional irreproducibility also occurred in the peak area of the samples, likely due to the insufficient buffering of the whole blood samples. These variations were not observed when derivatization was performed at pH 10. Thus, a 100 mmol/L sodium phosphate dibasic heptahydrate solution at pH 10 was used thereafter. Regarding the effects of diethylpyrocarbonate (DEPC), its concentration was not directly evaluated in this study, and a tentative value of 33 mmol/L was extrapolated from the literature.

### 2.3. Recovery and Matrix Effect

Recovery was determined as recovery% = [(C2 − C0)/C1] × 100, where C2 is the concentration of the analyte in the final solution after spiking with a known concentration of standard, C0 is the original concentration of analyte in the initial solution and C1 is the added known concentration of standard. Thus, the whole blood was spiked with four different concentration levels of the analytes within the linear range of 55–440 nmol/L. As presented in Table 1, for each concentration of analyte added, three replicates were analyzed and the recoveries, computed as above, were close to 100% at each concentration.

As C2 and C0 were calculated by the calibration curves constructed using a stable isotope-labeled version of ERY, d3ERY, recovery% was also used as a quantitative measure of the extent of matrix effect. A percent recovery close to 100% indicates that matrix effect is negligible. Conversely, values <100 or >100 indicate the suppression or enhancement of ionization, respectively. The mean percentage of recovery for the four spiked whole blood samples ranged between 99.8 and 105.6%. This suggests a limited matrix effect impact that can be minimized by d3ERY, thus ensuring the accuracy of the quantitative LC-MS/MS analysis. The potential for cross-talk between multiple reaction monitoring mode (MRM) functions was also evaluated using a plasma sample without the addition of IS, a blank solution containing the IS without the analytes, and a blank solution containing the IS and the analytes. Although the precursor ions for labeled and unlabeled ERY share the same product ion at *m*/*z* 95, no cross-talk between MRM functions was observed. The carry-over was investigated by injecting a blank solution at the start of the batch, at the transition from the standards to the samples, in the middle and as the last sample of the batch.

### 2.4. Linearity, Calibration Range, Precision, LOD and LOQ

The calibration curves were obtained by plotting the peak area ratios of analyte over internal standard versus concentration. Within the range from 35 to 1120 nmol/L, the calibration curves showed good linearity, with an average coefficient of determination (r^2^) of 0.999. Precision was evaluated in terms of repeatability (intraday precision) and intermediate precision (interday precision). Repeatability was assessed using data from the recovery experiments, while the interday precision was determined by assaying the same samples on 10 different days over a period of two weeks. The results, expressed as %RSD of measurements, were <7% for both repeatability and intermediate precision. LOD and LOQ were computed on five calibration curves using the following equations 3.3σ/S and 10σ/S, respectively, where σ is the standard deviation of the intercept and S is the slope of the calibration plot. The average LOD and LOQ were 10.30 and 31.21 nmol/L, respectively (an MRM chromatogram of LOQ is provided as Supplementary Materials).

## 3. Discussion

**Figure 3:**
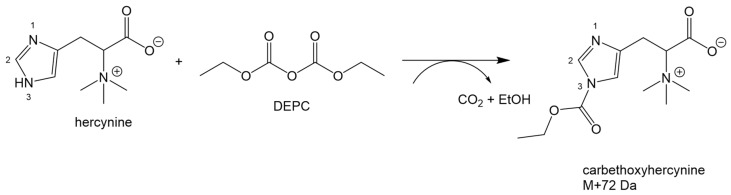
Derivatization of ERY by DEPC to form carbethoxyhercynine.

**Figure 4:**
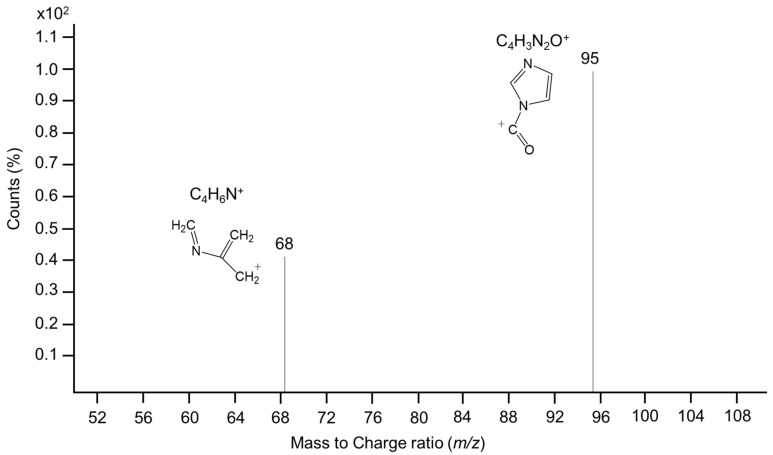
MS/MS spectra of carbethoxyhercynine *m*/*z* 270.28.

The development of an ad-hoc assay for ERY proved to be challenging. The betaine character of the molecule complicates its chromatographic separation on reversed-phase (RP) columns. Moreover, the optical features of ERY, primarily related to the imidazole ring, did not assist with measuring the low concentrations using a UV-Vis detector. In addition, the pre-treatment of the biological samples was important, as common procedures may affect the concentration of ERY. As ERY is a product of the redox activity of ERT, careful attention must be given to prevent the oxidation of ERT during sample handling. This is critical for measurements in whole blood where the lysis of red blood cells and protein removal steps can easily oxidize ERT, in a similar fashion to GSH [19]. As already observed with the latter, we found that filtration of the samples on micro concentrators, at a controlled temperature of 4 °C, was the easiest way both to preserve ERT/ERY concentrations, and to obtain the samples in a matrix that did not require further treatment [20] and that was, therefore, neutral with respect to the next analytical step. The undue oxidation of ERT was evaluated by leaving the samples at room temperature for an hour and then assessing any changes in ERY concentrations. Moreover, samples were spiked with ERT and, in both cases, no significant changes in ERY concentrations were observed. Regarding sensitivity, initially the derivatization of ERY with fluorescent labels was attempted, including 5-(bromomethyl)-fluorescein with 18-Crown-6 as a phase-transfer catalyst for the carboxyl moiety, and fluorenylmethyloxycarbonyl chloride, or dansyl chloride, for the imidazole ring. Derivatives were chromatographed on both HILIC and RP-columns, but attempts were unsuccessful due to difficulties in achieving a baseline separation of the peak of interest, derivatization, or low sensitivity to measure low concentrations of ERY. Field-amplified sample injection (FASI) capillary electrophoresis was also assessed to overcome both the chromatographic and sensitivity issues, however, no significant improvement was obtained compared to LC with HILIC and RP-columns. Subsequently, we worked to improve a previous CE-MS/MS method developed for the qualitative analysis of ERY in different human biological specimens [16]. For this purpose, a specific isotopically labeled internal standard for ERY, d3ERY, was used and a re-optimization of the MRM parameters, as well as the electrophoretic conditions, was performed. Although promising results were obtained, due to the complexity of the CE-MS/MS instrumentation, it was not possible to achieve a high-throughput method. Conversely, better results were obtained with the development of an LC-MS/MS method using an RP C18 column. This was accomplished by using a specific derivatization of the imidazole ring by DEPC, a well-known and powerful acylating agent of the imidazole ring of the histidine residues in proteins [21]. DEPC rapidly reacted with the imidazole ring of ERY, and LC-MS analysis revealed that, at the concentrations of DEPC used in this study two major compounds with a mass increase of 72 Da were formed from ERY. This was consistent with the formation of the carbethoxyhercynine *m*/*z* of 270.28, as seen in Figure 3, whose MS/MS spectra are shown in Figure 4. 

No further mass shifts due to histidyl overmodification were detected. Given the same mass-to-charge ratio and fragmentation pattern, a competitive acylation of the two nitrogen atoms in the imidazole ring would explain the two peaks observed at 4.6 and 9.5 min. Acylation is known to occur preferentially at position 3 on the nitrogen atom compared to position 1, and this would explain the difference in intensity of the two peaks. Regardless, derivatization with DEPC solved the chromatographic issues related to the betaine character of ERY, as well as sensitivity shortcomings. The derivatization of ERY enabled the use of an RP-column without ion pair reagents for the separation. Therefore, no signal suppression problems due to chromatography compromises had to be faced. In addition, probably by increasing the volatility in the ESI source, DEPC also enabled better ionization with an enhanced sensitivity and selectivity when compared to underivatized ERY. However, zwitterions are notoriously challenging to ionize for mass spectrometric detection, hence the derivatization strategy. Using the optimized LC-MS/MS method, the analysis of thirty real whole blood samples determined a mean ERY value of 178.5 ± 118.1 nmol/L. This value was lower than that reported in a previous article [16], where a semiquantitative approach was instead employed. It was also slightly lower than that obtained from Cheah et al. [18], although the age of the enrolled subjects (21–35 years) was much lower than the mean age in our study of 75 ± 11 years. Thus, as a positive correlation between ERT and ERY was described [18] and, given the reported decline in ERT levels with age [22], it is conceivable that ERY may also decrease with age. Furthermore, our data shows considerable variability in ERY concentrations. Like ERT levels, this could be indicative of between-subject differences in inflammatory status, or be explained by dietary habits [14], the physiology of red blood cells [23] or the expression of ETT mRNA [6]. As ERY is closely correlated with ERT, it is possible that the same mechanisms account for its variability.

## 4. Materials and Methods

### 4.1. Chemicals

Acetonitrile (ACN) of HPLC grade, sodium phosphate dibasic heptahydrate (Na_2_HPO_4_·7H_2_O), formic acid, sodium hydroxide (NaOH), diethylpyrocarbonate (DEPC), reagents for the synthesis of ERY and disposable ultrafiltration devices (Vivaspin 500 Micro Concentrators), were purchased from Sigma Aldrich (Milan, Italy). ERY was synthesized in four steps via the methyl ester derivative of l-histidine (data on the synthesis and NMR characterization are provided as Supplementary Materials). Deuterated ERY with a mass difference of 3 (d3ERY), was obtained from DBA Italia (Milan, Italy). High-purity water, obtained from a Millipore Milli-Q system, was used throughout the experiments (Merck Millipore, Milan, Italy). 

### 4.2. Solutions

Standard solutions of ERY and of the isotopically labeled internal standard (IS) d3ERY were prepared in ultrapure water as 5 mmol/L stock solutions and stored as 50 µL aliquots at −80 °C until use. Fresh working standard solutions were prepared on the day of the analysis by diluting the stock solutions with Milli-Q water. The stock solution of ERY was mixed with water to obtain the highest concentration of the calibration curve, 1 µmol/L. This solution was further diluted with water to obtain five concentrations of the analyte. Similarly, a fresh working standard solution for d3ERY was prepared in ultrapure water by a 1/200 dilution of the stock solution. Sodium phosphate dibasic heptahydrate, used as a buffer in the derivatization reaction, was prepared in ultrapure water at a concentration of 100 mmol/L and pH 9.3. DEPC, used as a derivatization reagent, was prepared in ultrapure water at a concentration of 33 mmol/L. 

### 4.3. Sample Treatment and Derivatization

A 200 µL-volume of whole blood was spiked with 1 μL of the working solution of internal standard and mixed thoroughly by vigorous vortex-mixing. Whole blood samples were then lysed, by hypotonic shock with cold water, in a sample/water ratio of 1:1.25. Whole blood (400 µL) was then filtered by centrifugation on Vivaspin 500 micro concentrators at 21,380× *g* for 30 min at 4 °C to remove proteins and molecules above 10 kDa. For derivatization, a 20 µL-volume of phosphate buffer and 40 µL of DEPC were added to 120 µL of clear filtered fluid. After vortex-mixing, samples were allowed to stand at room temperature for 5 min then analyzed by LC-MS/MS.

### 4.4. Participants to Study and Samples Collection

Thirty male volunteers with no medical history (aged 75 ± 11 years) were randomly selected for this study. After informed written consent was obtained, blood was collected by venipuncture in 5.4 mg K3EDTA vacutainer tubes. Without delay, a series of 300 µL aliquots of whole blood for each subject were collected and stored at −80 °C until use. The study was performed following the principles outlined in the Declaration of Helsinki and all procedures were approved by the local ethics committee. The protocol was approved by the ethics committee of Local Health Unit n°1 of Sassari (2262/CE).

### 4.5. LC Equipment, Chromatographic and Mass Spectrometer Conditions

The LC apparatus was a Waters system model Acquity UPLC equipped with a Waters Acquity UPLC tandem quadrupole mass spectrometer (TQD) (Waters Italia, Milan, Italy). The separation was achieved on a 100 mm × 4.6 mm Zorbax eEclipse pPlus C18 3.5 µm column by using a mixture of aqueous of 0.1% *v*/*v* formic acid and ACN (95:5) as a mobile phase, isocratically delivered at a flow-rate of 0.5 mL min^−1^. Chromatographic separation was carried out at 30 °C in an air-conditioned room at approximately 25 °C with samples held at 23 °C in the autosampler. The amount injected was 5 µL in full loop mode using a 5 μL sample loop. Column effluents were monitored by mass spectrometry multiple reaction monitoring mode (MRM). The run time of LC-MS/MS analysis was 10 min. The capillary voltage in the mass spectrometer was set at 2.5 kV while ESI source and desolvation temperatures were set at 150 and 500 °C, respectively. The desolvation gas (nitrogen) and collision gas (argon) were delivered at 600 L·h^−1^ and 0.5 mL·h^−1^, respectively. Mass spectral parameters for MRM transitions were optimized by IntelliStart in MassLynx V4.1 software (Waters Italia, Milan, Italy) by infusing 100 µmol/L of each compound at a flow rate of 20 µL min^−1^. Mass detection was accomplished in positive ion mode by MRM using precursor-product ion transitions *m*/*z* 270.28→95 and 273.21→95 for ERY and d3ERY, respectively. Cone voltage, collision voltage and dwell times were 28 V, 27 V, and 0.1 s for ERY, and 35 V, 25 V and 0.1 s for d3ERY, respectively.

## 5. Conclusion

Given that the redox activity of ERT might explain the presence of ERY in biological fluids, the measurement of ERY might serve to indirectly detect oxidative stress states or other biological processes involving ERT. This could be particularly advantageous as the peculiar redox behavior of ERT, which acts to rapidly restore ERT concentrations, can minimize potential fluctuations. Little is known about this betaine of the histidine in the body, which is in part due to the lack of reliable analytical methods and the difficulty in purchasing commercial standards. Following the in-house chemical synthesis of ERY, and considering different analytical and sample treatment strategies, an LC-MS/MS method was developed for the measurement of ERY levels in whole blood. The method is straightforward and does not require cumbersome procedures for sample purification and/or concentration, such as solid phase extraction and evaporation to dryness, but only an easy and fast derivatization by DEPC. Overall, the method proved useful in overcoming the issues related to the pre-analytical and analytical steps, allowing the reliable assessment of ERY concentrations for clinical and research purposes.

## Figures and Tables

**Table 1:** Recovery experiments. C0 and C2 are the mean of three replicates.

Sample	C0 (nmol/L ± SD)	C1 (nmol/L)	C2 (nmol/L ± SD)	Recovery%
Spike 1	119.5 ± 2.4	440	558.6 ± 15.6	99.8
Spike 2	119.5 ± 2.4	220	345.9 ± 9.0	102.9
Spike 3	119.5 ± 2.4	110	231.4 ± 11.7	101.7
Spike 4	119.5 ± 2.4	55	177.6 ± 9.8	105.6

## Supplementary Materials

The following are available online, ERY synthesis and NMR characterization, Figure S1: MRM chromatogram at LOQ level of 31.21 nmol/L.
