# Supplementary material for: Development of an LC–Tandem Mass Spectrometry Method for the Quantitative Analysis of Hercynine in Human Whole Blood
Source: Molecules. 2018 Dec 14;23(12):3326. doi: 10.3390/molecules23123326 (PMC6321398; doi:10.3390/molecules23123326)
Supplement: Supplementary file 1 [file molecules-23-03326-s001.pdf]

Synthesis of Hercynine, ERY

Histidine methyl ester dihydrochloride

Using a modified literature procedure [1], L-histidine (2.1 g, 13.5 mmol) was suspended in MeOH (40 mL) under a nitrogen atmosphere and $SOCl_2$ (1 eq., 1.0 mL) added dropwise at 0 °C. The solution was allowed to warm to room temperature and then refluxed for 6 hrs. The solution was cooled to room temperature and allowed to stir overnight. The solution was recharged with further $SOCl_2$ (1 eq., 1.0 mL) and refluxed for a further 6 hrs. The solvent was removed *in vacuo*, and the solids washed with EtOAc, Et_2O, and *n*-hexane to remove a yellow coloured impurity. The white powder was dried *in vacuo* to afford 3.0 g (92 %) of the product. ^1H NMR (400 MHz, D_2O, 26 °C): 8.67 (s, 1H), 7.45 (s, 1H), 4.52 (t, 6.8 Hz, 1H), 3.88 (s, 3H), 3.56-3.40 (m, 2H).

N,*N*-dimethyl histidine methyl ester

Using a modified literature procedure [2, 3], histidine methyl ester dihydrochloride (1.0 g, 4.1 mmol) was dissolved in MeOH (25 mL) under a nitrogen atmosphere. $NaCNBH_3$ [4, 5] (2.2 eq., 0.57 g, 8.3 mmol) and formaldehyde (2.5 eq., 10.3 mmol, 0.84 mL of 37 wt. % in H_2O) were added, and the solution stirred at room temperature for 4 hrs. The solvent was removed *in vacuo* in the fumehood (liquid nitrogen trap), and the product extracted from the residue with CH_2Cl_2 (3x 10 mL). The remaining solid was discarded, and the CH_2Cl_2 was removed *in vacuo* (as previously described) to afford an oil. The residue was purified by column chromatography (silica, 1% Et_3N in 20% MeOH/CH_2Cl_2) to afford 0.55 g (67 %) of a colourless oil. *Note: TLC of the chromatographed product in the absence of Et_3N in the mobile phase sometimes revealed two bands. In this case, the chromatographed product was repeatedly redissolved in CH_2Cl_2 (2-3 mL) and the solvent removed *in vacuo* (approx. 5x), until only a single band could be observed. ^1H NMR (400 MHz, $CDCl_3$, 26 °C): 7.53 (s, 1H), 6.83 (s, 1H), 3.71 (s, 3H), 3.49 (t, 7.3 Hz, 1H), 3.08-2.90 (m, 2H), 2.42 (s, 6H). ^{13}C NMR (100 MHz, $CDCl_3$, 26 °C): 171.57, 134.25, 131.65, 119.27, 67.15, 51.57, 41.67, 25.36.

Hercynine methyl ester

Using a literature procedure [2, 3, 6-8], *N*,*N*-dimethyl histidine methyl ester (231 mg, 1.2 mmol) was dissolved in MeOH (20 mL) under a nitrogen atmosphere, and the pH adjusted to approx. 8-9 (pH paper) using 30 % aqueous NH_4OH (60-150 µL depending on the batch). MeI (1.5 eq., 110 µL, 1.8 mmol) was added, and the solution stirred at room temperature for 24 hrs. The solvent was removed *in vacuo* in the fumehood (liquid nitrogen trap). CH_2Cl_2 (2-3 mL) was added to the residue and the solvent removed *in vacuo* as previously described (approx. 3x) to remove remaining MeI. The solid was recrystallised from MeOH/Et_2O, the supernatant removed, and CH_2Cl_2 was again added and removed *in vacuo* to afford 0.265 g (67 %) of a tacky white solid. ^1H NMR (400 MHz, DMSO-d_6, 26 °C): 7.80 (s, 1H), 7.03 (s, 1H), 4.53 (dd, J = 11.2, 3.4 Hz, 1H), 3.66 (s, 3H), 3.41 (dd, J = 14.0, 3.4 Hz, 1H), 3.26 (s, 9 H), 3.14 (m, 1H). ^{13}C NMR (100 MHz, DMSO-d_6): 167.01, 135.49, 131.16, 115.45, 73.23, 53.11, 51.86, 24.90.

Hercynine, **ERY**

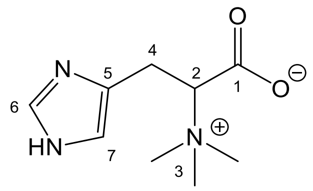

Using a modified literature procedure [3], hercynine methyl ester (424 mg, 1.25 mmol) was dissolved in 6M aqueous HCl (35 mL) and refluxed for 48 hrs. The aqueous HCl was removed by distillation under reduced pressure, after which the residue was recharged with fresh 6M HCl (35 mL), and refluxed for a further 24 hrs. The HCl solution was again removed by distillation under reduced pressure, and the residue recrystallised from MeOH/Et_2O. The resulting white powder was further purified [7] by flash column chromatography (silica, 10:2:1 MeOH:H_2O:30 % aqueous NH_4OH). The solvent was removed *in vacuo*, the powder redissolved in H_2O (Milli-Q, 2-3 mL) and filtered through a 0.22 µM PES membrane, and lyophilised to afford 60 mg (24 %) of a white powder used for experiments. ^1H NMR (400 MHz, D_2O, 26 °C): 7.70 (s, 1H, imidazole C^6H or C^7H), 6.99 (s, 1H, imidazole C^6H or C^7H), 3.91 (dd, J = 10.5, 4.8 Hz, 1H, C^2H), 3.32-3.19 (m, 11H, overlapping $N(C^3H_3)_3$ and C^4H_2). ^{13}C NMR (100 MHz, D_2O): 171.02 (C^1=O), 136.32 (imidazole C^6 or C^7), 131.88 (imidazole C^5), 116.38 (imidazole C^6 or C^7), 78.60 (C^2H), 52.13 ($N(C^3H)_3$), 25.29 (C^4H_2).

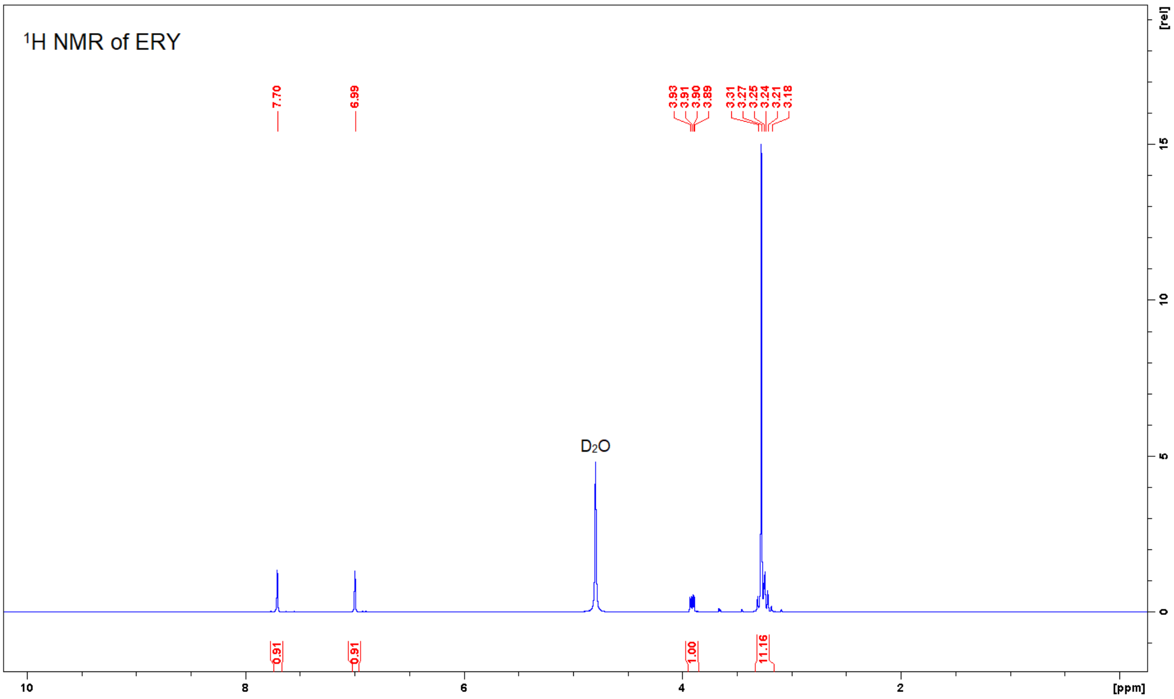
1H NMR of ERY
7.70
6.99
3.93
3.91
3.90
3.89
3.31
3.27
3.25
3.24
3.21
3.18
D2O
0.91
0.91
1.00
11.16
10
8
6
4
2
[ppm]
[rel]
15
10
5
0

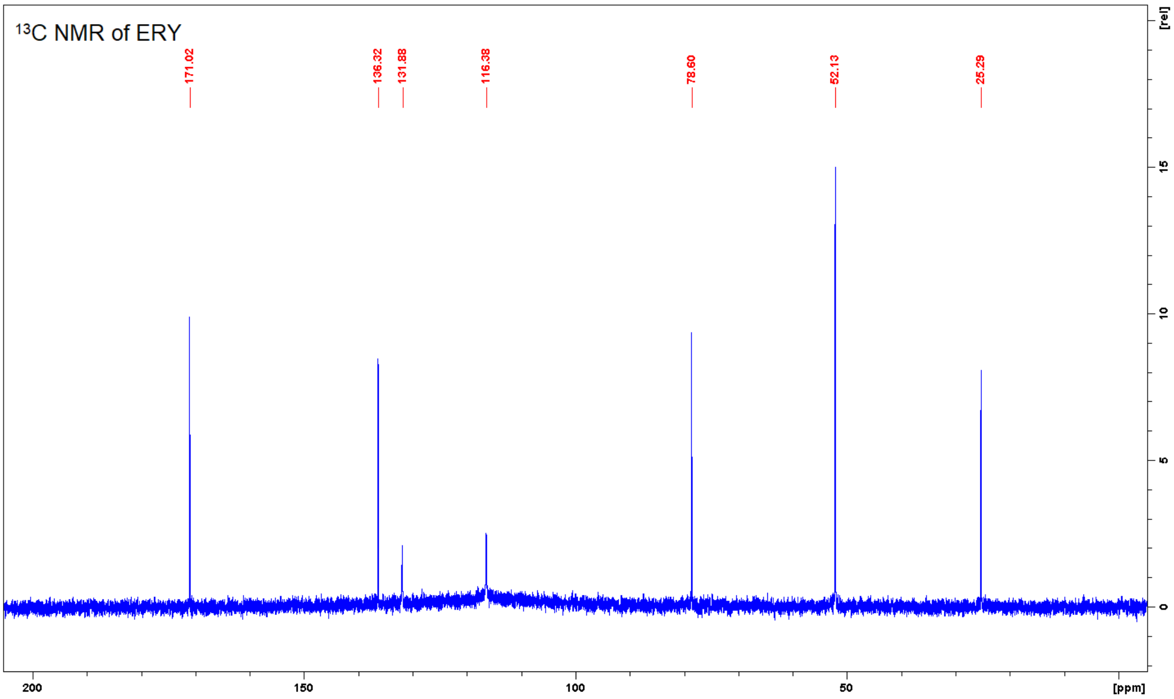
13C NMR of ERY
171.02
136.32
131.88
116.38
78.60
52.13
25.29
200
150
100
50
[ppm]
[rel]
15
10
5
0

COSY ERY

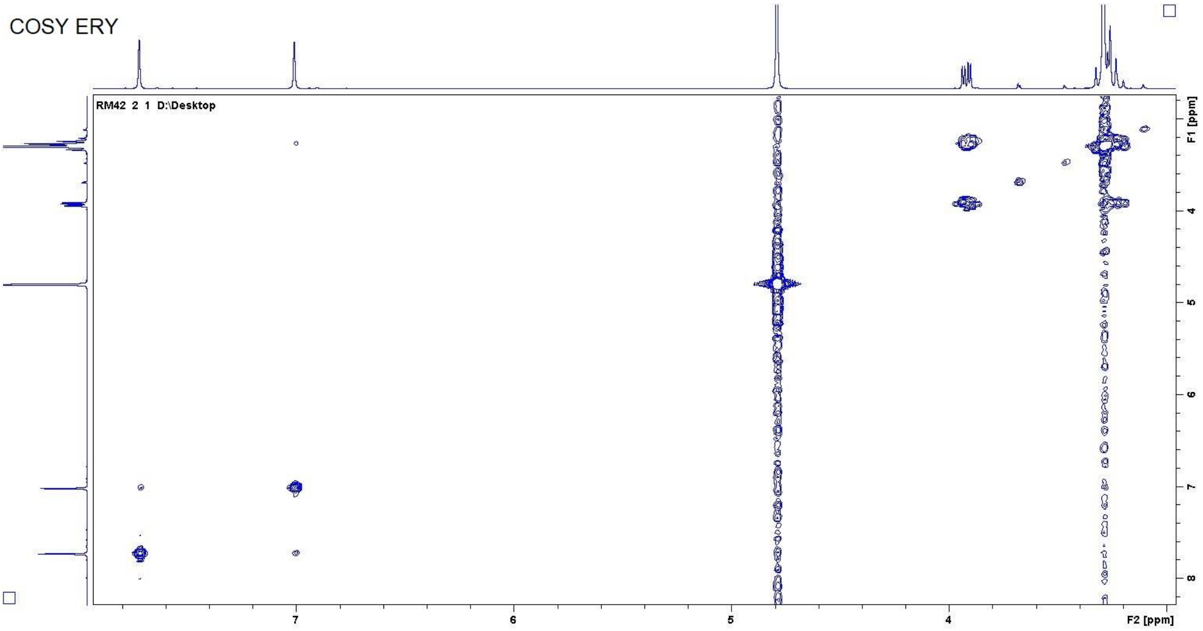

HMQC ERY

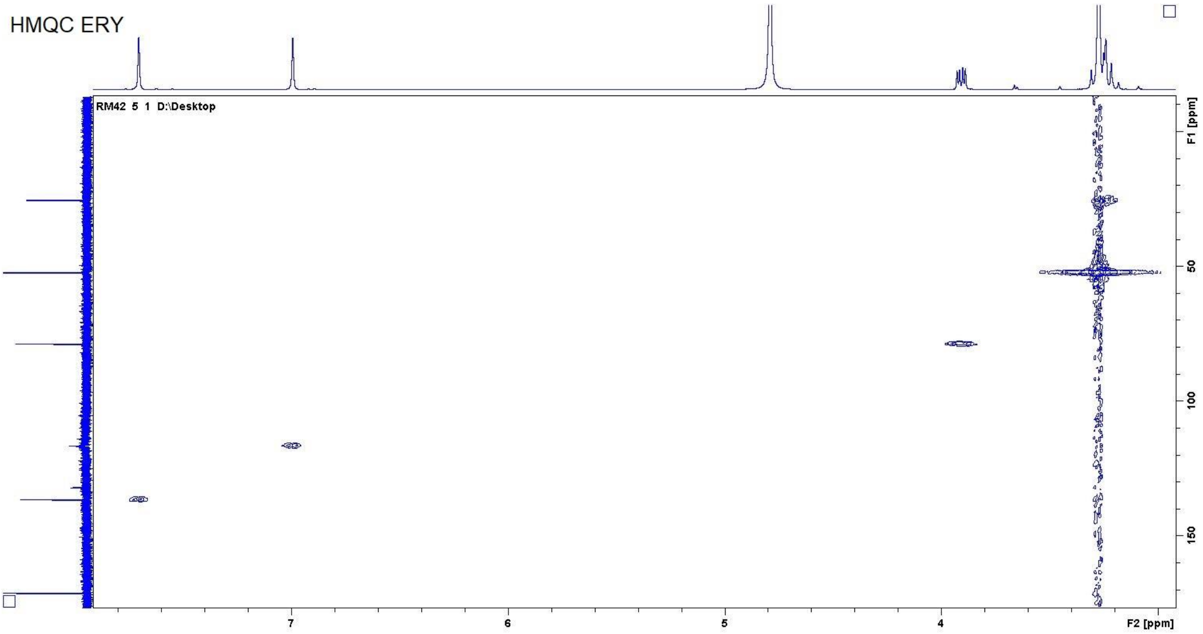

References

1. Turtaut, F., et al., MedChemComm, 2011. **2**(10): p. 995-1000.
2. Khonde, P.L. and A. Jardine, Organic & Biomolecular Chemistry, 2015. **13**(5): p. 1415-1419.
3. Khonde, L.P., *Synthesis of Mycobacterial Ergothioneine biosynthetic pathway metabolites*. 2013, Masters Thesis, Department of Chemistry, University of Cape Town, South Africa.
4. Jentoft, N. and D.G. Dearborn, *[50] Protein labeling by reductive alkylation*, in *Methods in Enzymology*. 1983, Academic Press. p. 570-579.
5. Naresh Chary, V., et al., Journal of Mass Spectrometry, 2015. **50**(5): p. 771-781.
6. Reinhold, V.N., Y. Ishikawa, and D.B. Melville, Journal of Medicinal Chemistry, 1968. **11**(2): p. 258-260.
7. Grigat, S., et al., Biochemical Pharmacology, 2007. **74**(2): p. 309-316.
8. Song, H., et al., Organic Letters, 2013. **15**(18): p. 4854-4857.

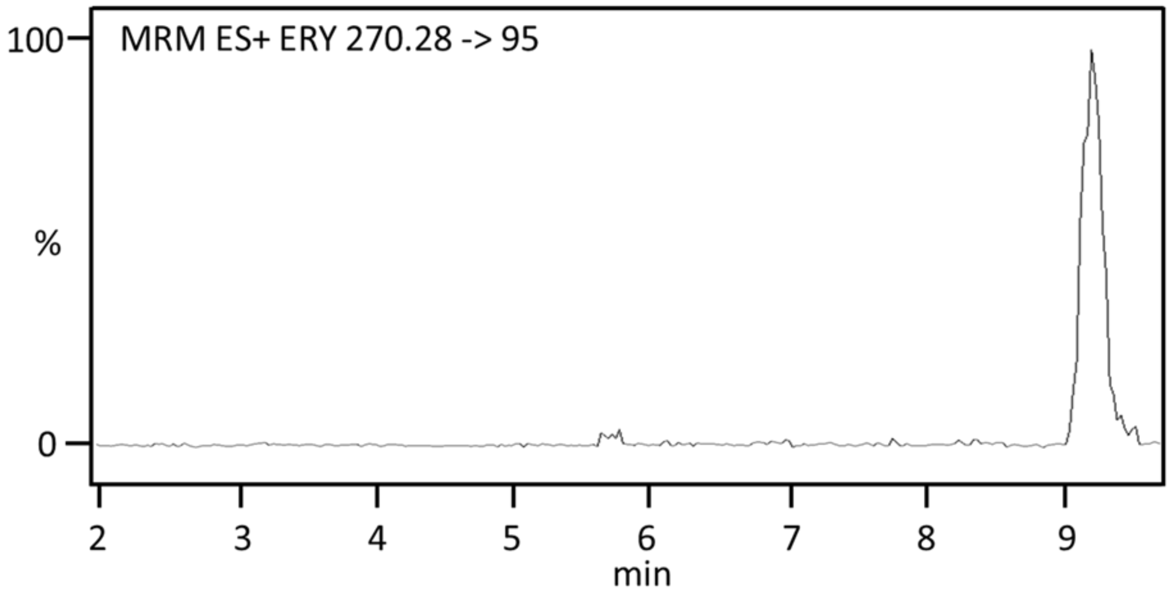

Figure S1. MRM chromatogram at LOQ level of 31.21 nmol/L
